# The Role of Alpha 2 Macroglobulin in IgG-Aggregation and Chronic Activation of the Complement System in Patients With Chronic Lymphocytic Leukemia

**DOI:** 10.3389/fimmu.2020.603569

**Published:** 2021-02-11

**Authors:** Naseba Naseraldeen, Regina Michelis, Masad Barhoum, Judith Chezar, Tamar Tadmor, Ariel Aviv, Lev Shvidel, Adi Litmanovich, Mona Shehadeh, Galia Stemer, Ety Shaoul, Andrei Braester

**Affiliations:** ^1^ The Institute for Medical Research, Galilee Medical Center, Nahariya, Israel; ^2^ Azrieli Faculty of Medicine, Bar Ilan University, Safed, Israel; ^3^ Institute of Hematology, Galilee Medical Center, Nahariya, Israel; ^4^ Hematology Unit, Bnai Zion Medical Center, Haifa, Israel; ^5^ The Ruth and Bruce Rappaport Faculty of Medicine, Technion, Haifa, Israel; ^6^ Department of Hematology, Emek Medical Center, Afula, Israel; ^7^ Hematology Institute, Kaplan Medical Center, Rehovot, Israel; ^8^ Faculty of Medicine, Hebrew University, Jerusalem, Israel; ^9^ Biochemistry Laboratory, Galilee Medical Center, Nahariya, Israel

**Keywords:** chronic lymphocytic leukemia, classical pathway, complement system, IgG-hexamers,, alpha 2 macroglobulin

## Abstract

Chronic lymphocytic leukemia (CLL) is the most common leukemia in adults in the western world. One of the treatments offered for CLL is immunotherapy. These treatments activate various cellular and biochemical mechanisms, using the complement system. Recently it was shown that the complement system in CLL patients is persistently activated at a low level through the classical pathway (CP). The mechanism of chronic CP activation involves the formation of IgG-hexamers (IgG-aggregates). According to recent studies, formation of ordered IgG-hexamers occurs on cell surfaces *via* specific interactions between Fc regions of the IgG monomers, which occur after antigen binding. The present study investigated the formation of IgG-hexamers in CLL patients and normal (non-malignant) controls (NC), their ability to activate complement, their incidence as cell-free and cell-bound forms and the identity of the antigen causing their formation. Sera from 30 patients and 12 NC were used for separation of IgG- aggregates. The obtained IgG- aggregates were measured and used for assessment of CP activation. For evaluation of the presence of IgG- aggregates on blood cells, whole blood samples were stained and assessed by flow cytometry. Serum levels of IgG- aggregates were higher in CLL and they activated the complement system to a higher extent than in NC. Alpha 2 macroglobulin (A2M) was identified as the antigen causing the hexamerization/aggregation of IgG, and was found to be part of the hexamer structure by mass spectrometry, Western blot and flow cytometry analysis. The presence of A2M-IgG-hexamers on B-cells suggests that it may be formed on B cells surface and then be detached to become cell-free. Alternatively, it may form in the plasma and then attach to the cell surface. The exact time course of A2M-IgG-hexamers formation in CLL should be further studied. The results in this study may be useful for improvement of current immunotherapy regimens.

## Introduction

Chronic lymphocytic leukemia (CLL) is the most common leukemia in adults in the western world. It affects the B-type lymphocytes in the bone marrow in 95% of the patients, and is characterized by increased numbers of monoclonal B-lymphocytes (>5 × 10^3^/µl) that express specific antigens (CD5, CD19, CD20, CD23) on their surface (1).

One of the treatments offered for CLL patients is immunotherapy which allows the immune system to identify cancer cells or train the immune system to fight the malignant B cells (2). The mechanisms involved by the immunotherapeutic monoclonal antibodies are complement dependent cytotoxicity (CDC), antibody dependent cell-mediated cytotoxicity (ADCC), lysosomal membrane permeability (LMP) (3) and phagocytosis (4, 5). The complement system is an ancient defense mechanism preceding adaptive immunity (6). Three complement pathways utilize the nine central proteins of this system (C1–C9): the classical (CP), alternative (AP) and lectin pathways. The interaction of the first component in each pathway with an activator leads to an ordered cascade activation, typical for each pathway (7). All the complement pathways eventually produce the Membrane Attack Complex (MAC, C5b-9), a cytolytic end product, which causes osmotic lysis of the pathogen/target cell (8). The complement system in CLL patients shows decreased levels of complement components (9), decreased CP activity and chronic activation at a low level (10, 11). The CP is involved in this chronic activity, and the decrease in CP activity was assumed to be due to fatigue (10).

The CP is initiated by the binding of C1q to the Fc regions of antigen-bound immunoglobulins type G or M (IgG or IgM) (7). Conformational changes in C1q lead to the activation of C1r which, in turn, activates C1s (6, 12). All these proteins are serine proteases that combine C1, the first component of the CP (13). Clq shows weak binding to the Fc regions of monomeric, non-aggregated IgG, while in the presence of aggregated/hexamerized IgG, that occurs after antigen binding to the Fab domain, the strength of the binding increases and the CP is activated efficiently (14, 15). The formation of the IgG-hexamers is not entirely understood. According to some studies the binding of the antigen on cells causes specific non-covalent interactions between Fc segments of IgG which lead to the arrangement of hexamers (16), suggesting that IgG can form ordered hexamers only on cell surfaces (15). Recently we have demonstrated the presence of aggregates in plasma of CLL patients, which are not bound to cell surfaces (cell-free) (17). The antigen stimulating the formation of these IgG-hexamers may have great importance for the chronic CP activation in CLL, and is described in this study for the first time.

## Materials and Methods

### Subjects

Blood samples were collected from 30 naïve CLL patients and 12 normal (non-malignant) controls (NC). Plasma and sera were separated and frozen at −80°C. The rest of the samples were analyzed in the biochemistry and hematology laboratories. Samples were carefully collected and handled as described (18) in order to avoid spontaneous complement activation. The study was approved by the Helsinki Committee (Institutional Review Board) of Galilee Medical Center, Nahariya, Israel. Patients were divided according to the detection of Ig-C5a (by Western blot analysis), a marker of chronic complement activity (10, 18).

### Separation of IgG-hexamers

#### Affinity Purification of Total IgG

Cell-free IgG were separated from sera/plasma using a commercial kit for total IgG extraction, based on affinity chromatography, according to the manufacturer’s instructions (Protein G HP SpinTrap™, GE Healthcare). The final stage of IgG separation included elution by a low pH (0.1 M glycine-HCl, pH 2.7), followed immediately by pH neutralization, according to the manufacturer’s instructions. The IgG separated by this kit includes all IgG molecules, i.e. monomers and hexamers. According to the manufacturer, other immunoglobulins (IgM etc.) are not separated by the kit. Fractions of non-IgG proteins produced during the early stages of the purification procedure, were stored at 4°C for later use. Some of the IgG-hexamer separations were repeated using a different method, the Melon Gel IgG Purification Kit (Thermo Fisher Scientific), which does not include any exposure of the samples to acidic conditions.

#### Size Selection

The total IgG fraction separated using the protein G kit was transferred to a filtration column with a cutoff of 1000 kDa (Vivaspin, Sartorius), and centrifuged for 3 min at 4K g at 4°C. Due to the high molecular weight of IgG-hexamers, >1000 kDa, they are retained on top of the column while monomeric IgG move to the bottom. Protein concentrations of the samples remaining on top and at the bottom of the filtration column were measured by Nanodrop (Thermo scientific), and were used, with the sample volumes, for calculation of total protein amount. Monomeric IgG were also stored at 4°C for later use.

### Protein Staining Methods

#### Silver Stain

To visualize the extracted IgG-hexamers, samples obtained after the filtration step were separated by SDS-PAGE and stained using a silver stain kit, (ProteoSilver™ Plus Silver Stain Kit- SIGMA). Results were documented using a gel imaging system (G-BOX, Syngene). To assure that the amount of the silver-stained proteins is sufficient for sequencing/mass spectrometry, the gels were de-stained and stained again with Coomassie blue (CB).

#### Western Blot Analysis

Sera/plasma of CLL patients were tested to identify specific proteins related to the complement system (the Ig-C5a complex, C5, IgG, α-2 macroglobulin [A2M]). Proteins were separated by SDS-PAGE with or without denaturation, transferred to a nitrocellulose membrane, and the studied protein was identified with the appropriate primary [anti human IgG (Sigma), anti human C5 (Quidel), anti human A2M (Gentex)], and the appropriate secondary antibodies. Signals were developed using an ECL kit (Immobilon^®^ Forte, Millipore) and documented using a gel imaging system (G-BOX, Syngene).

### Complement Activity

#### Complement Activity Assay

In order to assess CP activity, IgG-hexamers or in-vitro aggregated IgG (19), were incubated with diluted (1:20) normal sera as described (10). The IgG-hexamers obtained from 10µl serum were used for the assay. Non-IgG and monomeric-IgG, obtained during the IgG-hexamers separation procedure, were acetone precipitated (due to their high volume) and incubated with diluted normal serum, C1q depleted serum (Quidel) or Factor B depleted serum (Quidel). The activity was measured by the levels of MAC/sC5b-9, quantified using ELISA.

#### ELISA

To quantify the complement activation product MAC, an ELISA kit (sC5b-9 PLUS EIA, Quidel) was used according to the manufacturer’s instructions. The results were measured using an ELISA reader (CARIOSKAN LUX, Thermo scientific).

### Cell Analysis

#### Flow Cytometry

Whole blood samples were diluted to 10^4^/μl white blood cells (WBC), washed with PBS three times, and stained with fluorescent anti-CD19-PC7 (Beckman), anti CD45-APC (Beckman), anti-C1-FITC (Assaypro), anti A2M-PE (Assaypro), anti CD91-Per-CP-eF710 (Invitrogen, eBioscience), A2M-PerCP (Assaypro) and anti-GRP78 (anti-Bip, Cell Signaling Technology) antibodies. Anti-CD45 stains all the WBC while anti-CD19 marks B lymphocytes. C1 binds IgG only in its hexameric form and thus anti-C1 antibodies can detect cells to which IgG-hexamers and C1 are bound. Anti-CD91 and anti-78 kDa Glucose-Regulated Protein (GRP78) detect these A2M receptors on lymphocytes (20, 21). After incubation with the antibodies, the red blood cells were lysed with VersaLyse (Beckman). All incubations were performed at room temperature, in the dark, for 10 min. Cell staining was assessed by a Flow cytometer (NAVIOS, Beckman coulter).

#### Mass Spectrometry (Protein Sequencing)

The IgG-hexamers prepared from patients’ sera were separated by SDS-PAGE and silver stained (with ProteoSilver™ plus, Sigma). The heavy (γ) and light chains were identified by their molecular mass and high abundance. All other protein bands were excised from the gels and subjected to mass spectrometry at the Smoler Protein Research Center (Technion – Israel Institute of Technology, Haifa, Israel).

#### Monomeric IgG-Aggregation with A2M

The samples used for these experiments were the monomeric IgG fractions, obtained after IgG affinity columns and size selection (i.e. the sample at the bottom of the 1000 kDa Vivaspin column). Monomeric IgG concentrations were measured by Nanodrop and 7.5 µg from each sample were incubated with 15.8µg of A2M. These protein amounts give a molar ratio of 1:1 for the IgG vs. the A2M dimer, namely one monomeric IgG molecule to one A2M dimer. As a control, monomeric IgG was incubated with human serum albumin (HSA) at a molar ratio of 1:1, or without any protein. All incubations were performed in 100µl PBS for 2hr at room temperature in glass test tubes (as A2M adsorps to non-glass surfaces) (22). After the incubation, samples were used for size selection using the 1,000 kDa Vivaspin columns (Sartorius). The volumes of the samples obtained on top and bottom of the columns were measured, and the IgG concentrations were quantified using a human-IgG ELISA kit (Invitrogen).

### Statistical Analysis

Data were analyzed by Kolmogorov-Smirnov, Kruskal-Wallis and by Mann-Whitney tests, as appropriate. P < 0.05 was considered significant.

## Results

### Characteristics of the Subjects’ Groups

Clinical parameters of patients and NCs are shown in **Table 1**. WBC values were significantly higher in patients than in NC. Platelets were significantly higher in NC than in patients, although they were within the normal range. No significant differences were observed in all other parameters.

**Table 1:** Characteristics of the subjects’ groups.

Clinical Parameters (normal range)	CLL Patients	Normal Controls
n	30	12
Gender (male/female)	14/16	5/7
Age (years)	69.4 ± 10.9	59.7 ± 10.1
Serum C3 mg/dl (82–158)	109.7 ± 23.9	125.4 ± 21.5
Serum C4 mg/dl (15–35)	28.2 ± 8.8	29.3 ± 2.3
Cholesterol mg/dl (<200)	158.1 ± 35.2	203.6 ± 80.9
Triglycerides mg/dl (<150)	144.3 ± 78.5	177.1 ± 72.9
HDL mg/dl (>40)	37.8 ± 13.6	43.7 ± 9.6
LDL mg/dl (<100)	91.3 ± 38.7	112 ± 52.4
Non-HDL chol. mg/dl (<130)	120.1 ± 31.2	160.1 ± 80.5
Platlet X10e3/µl (130–400)	164.17 ± 75.9*	272.5 ± 98.3
WBC X10e3/µl (4–10)	33.3 ± 69.3*	7.4 ± 2.8
HGB g/dl (13–18)	13 ± 2.01	13.6 ± 1.1
Neutro abs X10e3/µl (1.5–8)	4.6 ± 2.1	4.7 ± 2.4

All values are given as mean ± SEM.

*indicates significant p value (p < 0.05).

### The Levels of Cell-Free IgG-Hexamers in Patients and NC

**Figure 1:**
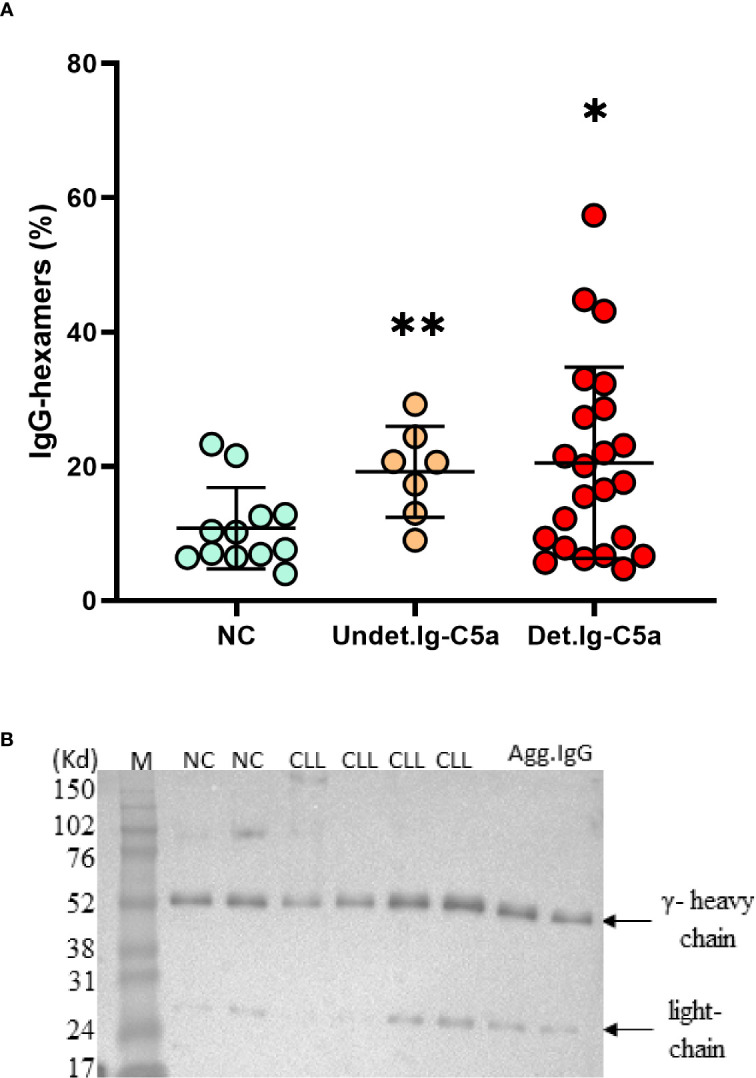
Cell free IgG-hexamers patients and NC subjects. IgG-hexamers were purified from sera/plasma of CLL patients and NC subjects. **(A)** The percentage of IgG-hexamers were calculated. Detectable Ig-C5a (Det.Ig-C5a), n = 23, undetectable Ig-C5a (Undet.Ig-C5a) n = 7, NC n = 12. *, ** indicates significant p values (p < 0.04, 0.004, respectively) vs NC. **(B)** The samples were studied by Western blot using anti-IgG antibodies (representative results), equal protein amounts (10µg) were loaded in each lane. In vitro aggregated commercial IgG (Agg.IgG) was separated in parallel as a control. The heavy and light chains of the IgG are indicated by arrows.

Protein G columns and 1,000 kDa filtration columns were used for separation of IgG-hexamers, and the percent of IgG-hexamers were calculated. The percent of IgG hexamers in the NC samples was 9.8 ± 5% of the total IgG while significantly higher percentage was observed in patients with and without chronic complement activation [according to the detection of Ig-C5a (10)], showing 19.53 ± 3% and 20.26 ± 4%, respectively (**Figure 1A**). The presence of IgG-hexamers was also verified by another separation method (The Melon Gel Kit), which does not include an elution step of the IgG by a low pH. The percentage of IgG-hexamers obtained by the Melon Gel Kit and the Protein G columns showed significant correlation (**Supplementary Data Sheet 1**). The identity of the separated IgG-hexamers was verified by Western blot using anti-human IgG antibodies (**Figure 1B**).

### IgG-Hexamers Contribute to the Chronic CP Activation in CLL Patients Serum

**Figure 2:**
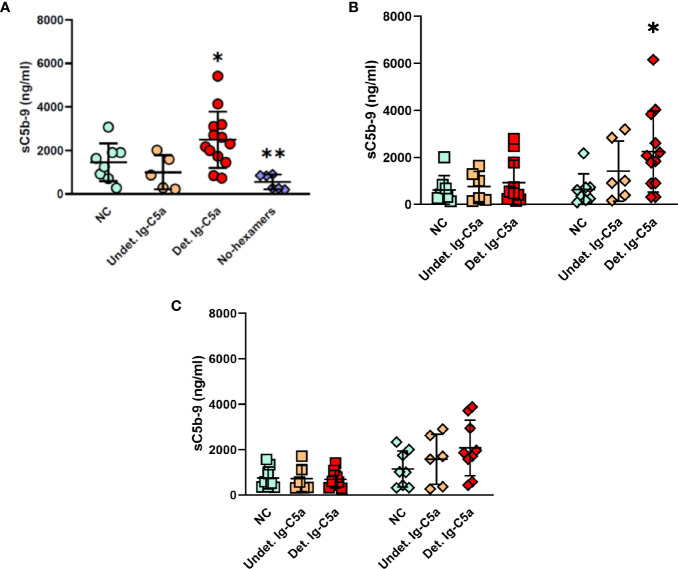
Activation of the complement system by IgG-hexamers. Complement activity was measured in normal serum after incubation with IgG-hexamers from NC and patients **(A)**. Serum samples incubated with buffer were used as a negative control. Proteins that did not bind to the protein G columns (non-IgG proteins, ◊), and non-hexameric IgG (monomeric IgG, **□**) were used for complement activation in C1q depleted serum **(B)** and factor B depleted serum **(C)**. Activation was followed by the levels of sC5b-9. Detectable Ig-C5a (Det.Ig-C5a) n = 12, Undetectable Ig-C5a (Undet.Ig-C5a) n = 6, NC n = 8. *, ** indicates significant p values (p < 0.05, 0.005, respectively) compared to NC and No-hexamers.

To assess the CP activation capacity of the IgG-hexamers, the CP was activated in normal serum by addition of the IgG-hexamers’ preparations, and activity was assessed by the levels of the sC5b-9 produced (**Figure 2**). IgG-hexamers from patients with detectable Ig-C5a activated complement to a higher extent than those from NC plasma. IgG-hexamers from patients with undetectable Ig-C5a did not show similar results (**Figure 2A**). Non-IgG proteins (proteins that did not bind to the protein G columns) were able to activate complement in C1q-depleted serum (**Figure 2B**) suggesting activation *via* the AP. The data obtained using factor B depleted serum (**Figure 2C**) support AP activation. Non-hexameric (monomeric) IgG samples from patients or NC did not induce complement activation.

### Cell-Bound IgG-Hexamers Are Detected on B Cells

**Figure 3:**
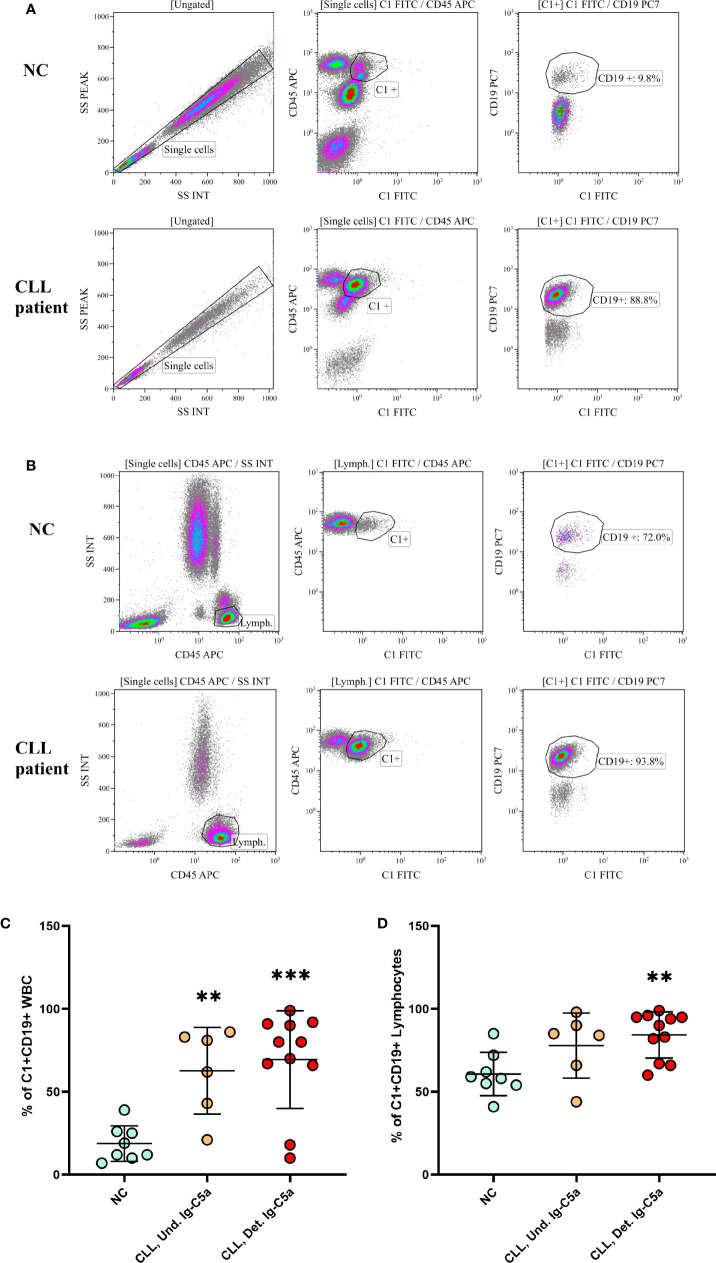
IgG-hexamers on B cell surface. Blood samples from CLL patients and NC were stained with fluorescent antibodies against CD45, CD19 and C1, and tested in a flow cytometer. representative results are shown **(A, B)**. The results were gated on WBC **(A, C)** or on lymphocytes **(B, D)**. Detectable Ig-C5a n = 11; Undetectable Ig-C5a n = 6; NC n = 8. **, *** indicate significant p values (p < 0.01, 0.001, respectively) compared to NC.

Fresh blood samples from NC and CLL patients were stained with fluorescent antibodies against CD45, CD19 and C1 and tested in a flow cytometer in order to assess IgG-hexamers that are present on cells’ surfaces. The results showed that the anti-C1 antibody stained WBC (CD45^+^) and particularly B cells (CD19^+^). In NC the C1+CD19+ staining was 20 ± 10% and 65 ± 8% in patients, when gated on WBC (**Figure 3A**, representative results and **Figure 3C**). In order to overcome the increase in B cell population in the patients, analysis was performed again after gating on lymphocytes. The percent of C1+CD19+ cells was significantly higher in patients, showing 95 ± 3%, compared to only 60 ± 20% in NC (**Figure 3B**, representative results and **Figure 3D**). The results were negatively correlated with the levels of cell-free IgG-hexamers (p < 0.03). This observation suggests that cell-free IgG-hexamers are in equilibrium with the cell-bound hexamers found on B-cell surfaces.

### Identity of the Antigen That Is Causing the Hexamerization

**Figure 4:**
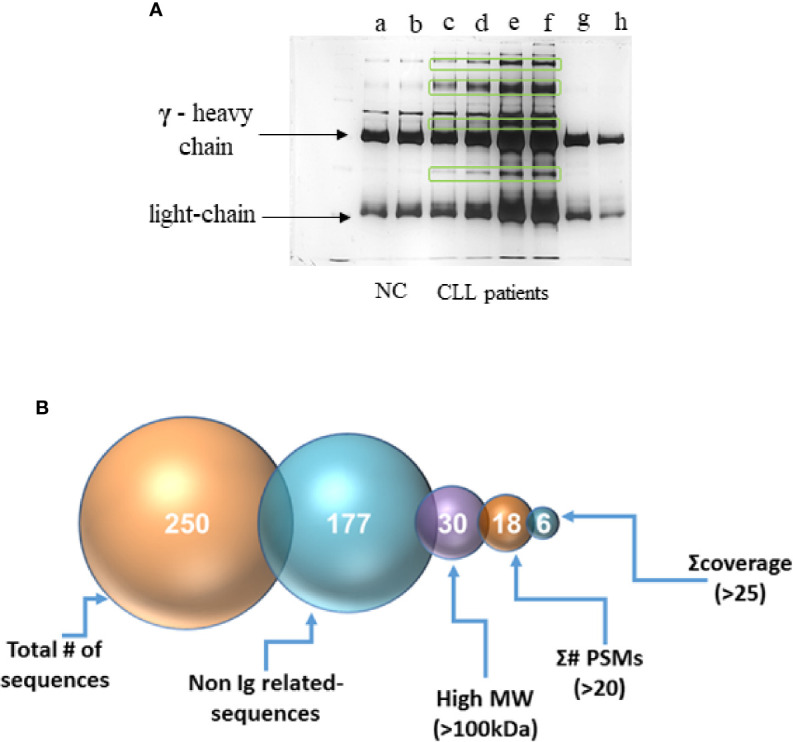
Separation of the IgG-hexamer samples. **(A)** Samples of NC (a, b), CLL patients (c–f) and commercial IgG (g, h) were separated and silver stained. Heavy (γ) and light chain are indicated by arrows. Additional proteins besides heavy and light chains are marked in frames. **(B)** The process of selection of the resulting sequence data included elimination of all the IgG-related sequences, low molecular mass peptides, sequences with a total number of identified peptide sequences (peptide spectrum matches-#PSMs)<30, and sequences with coverage<25.

**Figure 5:**
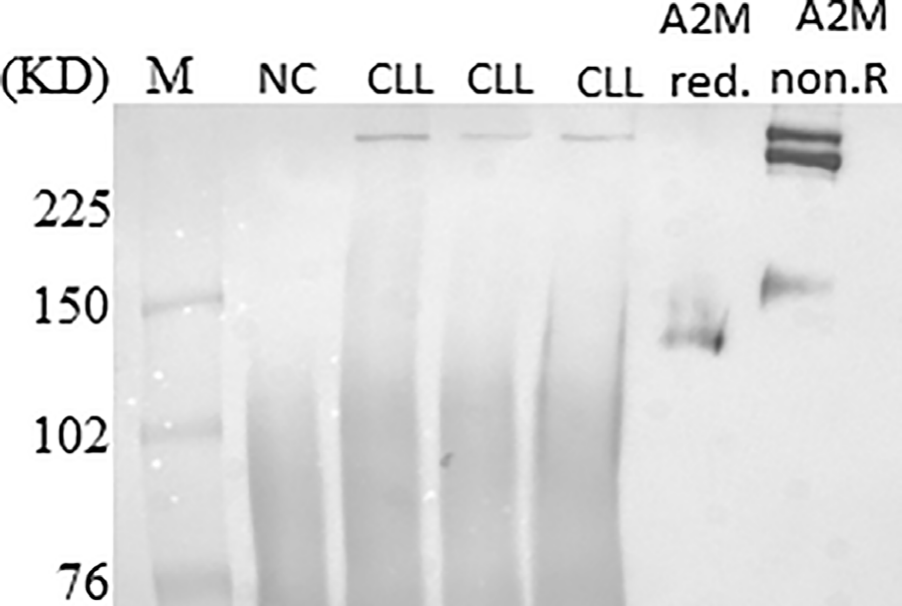
A2M presence in IgG-hexamers samples. A2M was identified in IgG-hexamers samples by Western blot using anti-A2M antibodies (representative results). Purified commercial A2M, reduced (red.) and non-reduced (non.R) were used as a positive control.

Ten µg of the IgG-hexamer samples were used for SDS-PAGE and silver staining. The results showed additional proteins besides the heavy and light chains of the IgG (**Figure 4A**). These were excised from the gel and subjected to mass spectrometry. After elimination of all the IgG-related sequences, low molecular mass peptides, sequences with a total number of identified peptide sequences (peptide spectrum matches-#PSMs)<30, and sequences with coverage<25, the results indicated six proteins suspected as the antigens that may cause the IgG-hexamerization (**Figure 4B**). One of these candidates was found to be the A2M. The raw sequencing data of the A2M identified peptides can be accessed in the Dryad database under the accession number https://doi.org/10.5061/dryad.tmpg4f4xg. A2M was further studied by Western blot using anti-A2M antibody to verify the presence of A2M in the IgG-hexamer preparations. The Western blot results indicated A2M presence in IgG-hexamer samples of CLL patients (**Figure 5**) and very low or no A2M signal in NC samples, indicating its participation in the hexamer structure. A2M showed a MW of ~360 kDa, similar to that of the purified commercial A2M that was used as a positive control (**Figure 5**).

**Figure 6:**
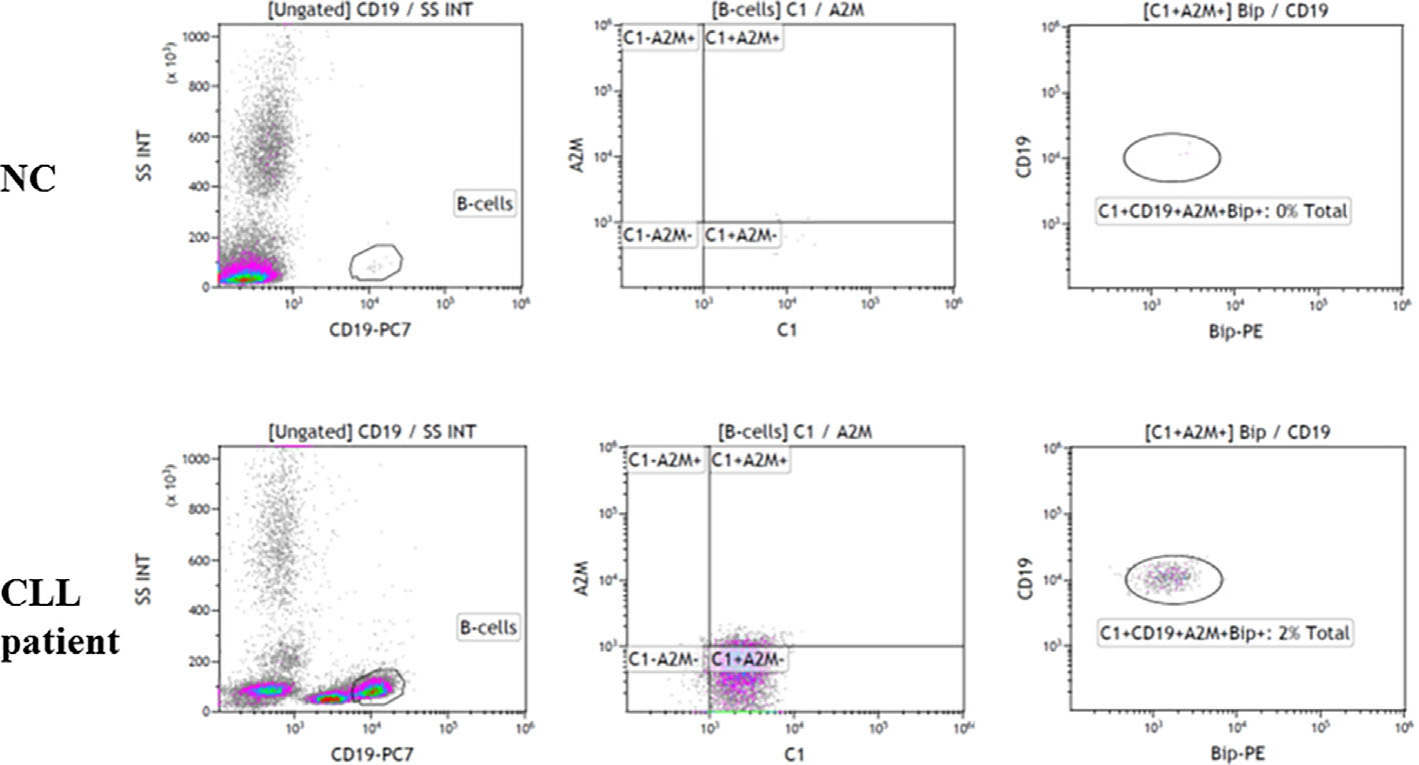
Association of the A2M-IgG-hexamers with B lymphocytes. Blood samples from CLL patients and NC were stained with fluorescent antibodies against CD45, CD19, C1, A2M and GRP78 (Bip) and tested in a flow cytometer. Representative results are shown.

The association of the A2M-IgG-hexamers with B lymphocytes was studied by flow cytometry. Fresh blood samples from NC and CLL patients were stained with fluorescent antibodies to A2M, C1 and two A2M receptors, the CD91 (data not shown) and GRP78. High A2M+GRP78+ staining in C1+CD19+ cells supports the presence of A2M-IgG-hexamers on B-cells in addition to the cell-free form (**Figure 6**). The data suggest that the A2M-IgG-hexamers are attached to B-cells by binding the GRP78 (**Figure 6**).

### Monomeric IgG Form Hexamers *in-Vitro* After Incubation with A2M in a Cell-Free Environment

**Figure 7:**
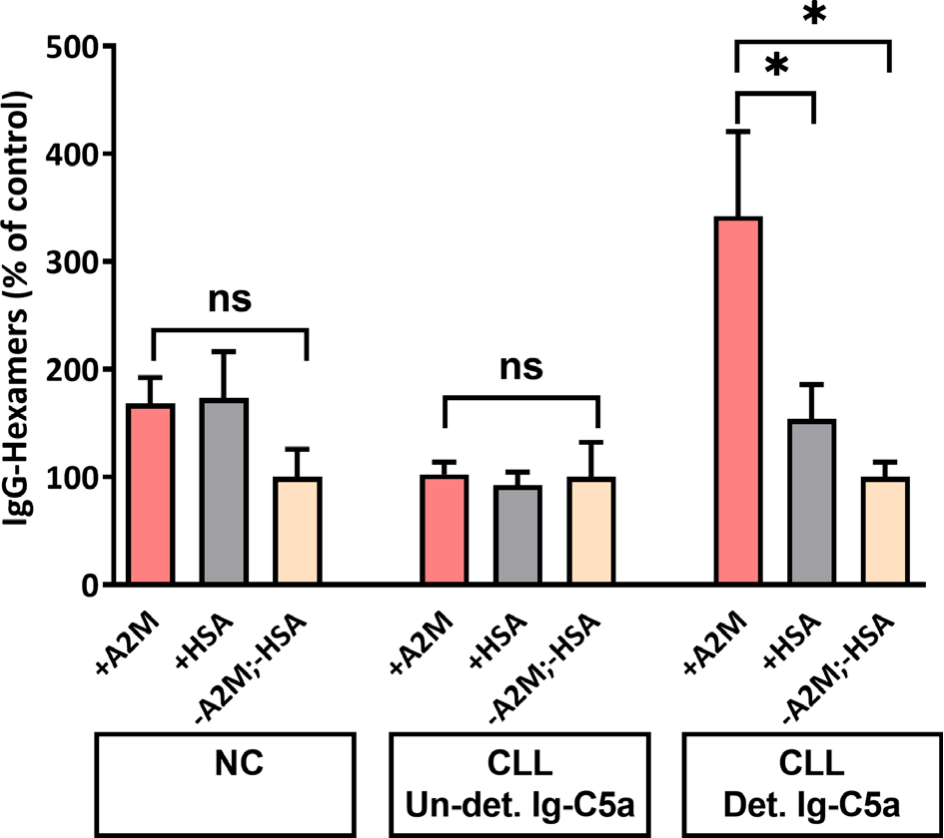
IgG-hexamer formation after in-vitro incubation of monomeric IgG with A2M. Monomeric IgG were incubated with purified commercial A2M, HSA or PBS and the generated IgG-hexamers were separated. IgG were quantified by ELISA and the percent of IgG-hexamers was calculated. *, ns indicate significant (< 0.05) and non-significant p values, respectively.

Our working assumption was that IgG molecules which bind A2M are those that form hexamers/aggregates. We assessed this assumption by incubation of total serum monomeric IgG with purified commercial A2M, followed by separation and measurement of the generated hexamers. The results shown in **Figure 7** indicate a significant increase in IgG-hexamer generation after incubation of A2M with monomeric IgG from patients with detectable Ig-C5a. Hexamer generation without addition of A2M was minimal, only 4.9% to 7.8% and without significant differences between the subject groups. A non-significant increase in IgG-hexamers was found in the NC group, and no increase was observed in patients with undetectable Ig-C5a (**Figure 7**). Also, no hexamerization was observed after incubation of IgG with a different protein, HSA. The results indicate the presence of anti-A2M antibodies in sera of patients with detectable Ig-C5a (chronic complement activation) and support the potential formation of hexamers in a cell-free environment, i.e. in the plasma.

## Discussion

Our study focused on characterization of the IgG-hexamers that are related to chronic CP activation in CLL. IgG-hexamers normally bind C1, the first component of the CP, and activate a cascade of complement proteins until C5b-9 is formed. The high level of these cell-free hexamers in patients has a key role in chronic CP activation, leading to the “weariness” of this pathway as well as to an increase in the formation of Ig-C5a complex and in the levels of other complement activation markers (17). IgG-hexamers are mainly formed after antigen binding that leads to non-covalent Fc-Fc interactions (23). We assumed that in CLL patients a particular antigen (or antigens) causes increased formation of IgG-hexamers, as shown by their high percentages in the patients’ plasma. In this study, the antigen causing hexamerization was found to be A2M.

The IgG-antigen aggregates (immune complexes) exist on the surface of malignant B cells and also as a detached, cell-free, form. The percentage of both cell-free and cell-bound IgG-hexamers is significantly higher in patients than in NC. The increased ability of these hexamers/aggregates to activate the CP supports their potential role in the chronic activation of the CP in CLL.

It is important to mention that in this study we show that natural IgG aggregates exist in complex with A2M, but we did not demonstrate the hexameric rings. Thus, the complexes formed between IgG and A2M may potentially involve Fab-mediated clustering of dimeric A2M, in addition to the known Fc : Fc interactions. In this case, large aggregated networks may be formed, and these aggregates may include complexes including more than six IgG molecules (17). These aggregates are very potent in complement activation and in depleting the complement system in CLL patients.

During normal physiological conditions, AP dominates activation of the complement system in plasma due to the spontaneous hydrolysis of complement component C3 (24). This hydrolysis can be hastened through some biological interfaces that include lipid complexes (8). Moreover, non-IgG proteins (from CLL patients with chronic complement activation) may also activate the AP, as suggested in this study by the data obtained using C1q depleted serum.

A previous study showed decreased expression of CD19 molecules on B-cells from CLL patients compared to normal B-cells (25). This information supports the significance of our findings on the differences in C1+CD19+ cell populations between patients and NCs and suggests that our findings are truly due to a higher presence of IgG-hexamers on B cell surface in the patients, rather than due to increased expression of CD19.

One or several of the additional non-IgG proteins that were found in the IgG-hexamer preparations was the antigen/s causing the hexamerization. Mass spectrometry (protein sequencing) analysis indicated that A2M could be the antigen. A2M is formed by the assembly of four 180-kDa subunits into two disulfide-linked dimers, which noncovalently associate to complete the tetrameric structure of A2M (26). However, several studies showed that A2M is present in the circulation in either a dimeric or tetrameric form (27, 28). A2M can bind through the CD91 receptor to T cells (20), and one study showed that A2M could also be expressed on B lymphocytes (29). Another receptor for A2M is GRP78, a member of the HSP70 gene family (30), whose levels are elevated in hypoxia and starvation conditions, allowing it to function as a shield for solid tumors in these cases (31). Studies have shown that GRP78 is mainly expressed on B cells compared to T cells and its levels are significantly higher in patients with CLL than in NC (32).

Both the Western blot and flow cytometry analyses confirmed that A2M was the antigen causing IgG hexamerization. The data suggest that A2M-IgG-hexamers do not necessarily form on the surface of B cells as they can also form in plasma before binding to the cells *via* A2M receptors. Antibodies that react against A2M may be part of a more general autoimmune phenomenon which is established occasionally in CLL patients (33, 34).

In hematologic malignancies, C activation can have both protective properties as well as tumor growth promoting effects, both direct and indirect (35). For example, hematological tumor cells show enhanced expression and surface binding of C regulators such as Factor H, Factor H like protein 1 (FHL-1), Factor H related protein 1 (CFHR1), FHR-4, FHR5, and C4b binding protein (C4BP). These C regulators further display the cofactor activity, which function together with factor I to block C activation at the level of C3 convertase, and lead to C evasion (35). Similarly to these soluble regulators, the membrane-bound C inhibitors CD46, CD55 and CD59 are up-regulated in various primary tumors and tumor lines to evade the C attack (35). Also, the soluble form of the C receptor 1 (CR1), expressed on most leukocytes membranes, is shed into the plasma, where it functions as a powerful C inhibitor. This represents another possible C evasion strategy utilized by leukemia cells (35).

Although our findings regarding the A2M-IgG-hexamers are novel, the factors that control the hexamerization process and the sequence of their formation in CLL plasma and on B-cells have not yet been revealed and need further clarification. For the benefit of the patients, the results may be useful for improvement of current immunotherapy treatments, for example if A2M-IgG-hexamerization and the resulting chronic CP activation can be inhibited, and thus help the complement system attain maximal capacity. Alternatively, measurements of this protein or other complement activity markers can help physicians to identify patients who may be less responsive to immunotherapy. For these patients, other therapeutic options should be offered.

## Data Availability Statement

The raw sequencing data of the A2M identified peptides can be accessed in the Dryad database under the accession number https://doi.org/10.5061/dryad.tmpg4f4xg.

## Ethics Statement

The studies involving human participants were reviewed and approved by Institutional Review Board of Galilee Medical Center, Nahariya, Israel. The patients/participants provided their written informed consent to participate in this study.

## Author Contributions

NN: data curation, methodology. RM: conceptualization, data curation, data analysis, methodology, project administration, supervision, validation, writing – original draft, writing – review and editing. MB: project administration, supervision. JC: data curation, data analysis, methodology. TT: conceptualization, resources. AA: resources. LS: resources. AL: resources. MS: methodology. GS: resources. ES: methodology. AB: supervision, resources, project administration, methodology, funding, conceptualization, review and editing. All authors contributed to the article and approved the submitted version.

## Funding

The study was funded by the Health Corporation of Galilee Medical Center.

## Conflict of Interest

The authors declare that the research was conducted in the absence of any commercial or financial relationships that could be construed as a potential conflict of interest.
