# Supplementary material for: The Role of Alpha 2 Macroglobulin in IgG-Aggregation and Chronic Activation of the Complement System in Patients With Chronic Lymphocytic Leukemia
Source: Front Immunol. 2021 Feb 11;11:603569. doi: 10.3389/fimmu.2020.603569 (PMC7905172; doi:10.3389/fimmu.2020.603569)
Supplement: Supplementary file 1 [file DataSheet_1.pdf]

Supplementary data

In this study, cell-free IgG were separated from sera/plasma using a commercial kit for total IgG extraction, based on affinity chromatography (Protein G HP SpinTrapTM, GE Healthcare). The IgG separated by this kit includes all IgG molecules, i.e. monomers and hexamers, and no other immunoglobulins (IgM etc., according to the manufacturer). The final stage of IgG separation includes elution by a low pH (0.1 M glycine-HCl, pH 2.7), followed immediately by pH neutralization, according to the manufacturer's instructions. This step may lead to some IgG aggregation due to the exposure to an acidic pH. Thus, some of the IgG-hexamer separations were repeated (in the same samples) using a different method, the Melon Gel IgG Purification Kit (Thermo Fisher Scientific), which does not include any exposure of the samples to acidic conditions.

The percentage of IgG-hexamers obtained by the Melon Gel Kit and the Protein G columns showed significant correlation, as shown in the supplementary figure S1. This observation supports the presence of IgG-hexamers in some CLL patients, as was suggested by the data obtained with the Protein G affinity chromatography.

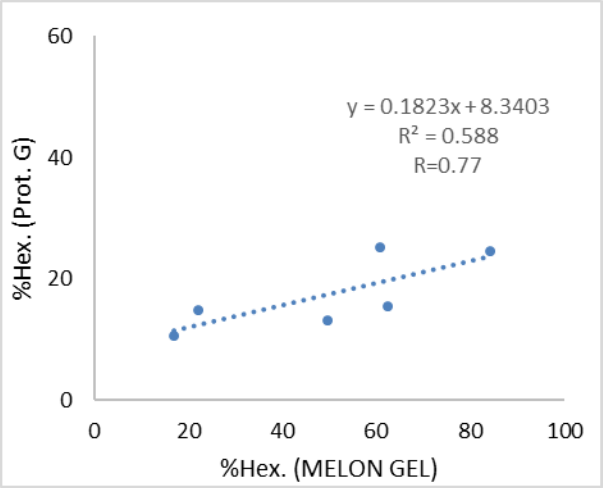

S1 Figure. ***Correlation between the percentage of IgG-hexamers obtained by the Melon Gel Kit and the Protein G columns.***

The percentage of IgG-hexamers (%Hex.) was obtained in the same samples by two methods: the Melon Gel Kit and the Protein G columns (Prot.G). The correlation between %Hex. obtained by the two methods is shown.
